# Antecedents of Webrooming in Omnichannel Retailing

**DOI:** 10.3389/fpsyg.2020.606798

**Published:** 2020-11-30

**Authors:** Kristina Kleinlercher, Marc Linzmajer, Peter C. Verhoef, Thomas Rudolph

**Affiliations:** ^1^Institute of Retail Management, University of St.Gallen, St. Gallen, Switzerland; ^2^Department of Marketing, Faculty of Economics and Business, University of Groningen, Groningen, Netherlands

**Keywords:** webrooming, omnichannel, search behavior, shopping motivations, customer spending

## Abstract

Although webrooming has become common practice in omnichannel consumer behavior, only a few empirical studies have managed to shed light on the phenomenon. With this research work, we aim to investigate important antecedents of webrooming. We base our conceptual framework on anticipated utility theory and expect that customers’ anticipated utility from using the physical store versus the online store for purchase can be predicted by four groups of antecedents: psychographic variables, shopping motivations, channel-related variables, and product-related variables. With the help of a data set from a large cross-national online survey in which 1497 customers reconstruct their last purchase journey, we differentiate webroomers from pure online shoppers. In addition, we disentangle customers who used retailer-owned, competitor-owned, and independent touchpoints along the search and purchase phase of the customer journey in order to characterize webroomers in an omnichannel context and assess their prevalence in different countries and industries. Our insights on the characteristics and antecedents of webrooming help retailers to detect and better understand the psychology behind the webrooming phenomenon from a consumer perspective in an omnichannel retailing environment. In addition, results from our exploratory analysis on the positive association between webrooming and customer spending contribute to research and practice by providing first evidence on the economic value of webrooming.

## Introduction

The rise of the internet and advances in information technology provide customers with a myriad of new touchpoints to interact with retailers, their competitors, manufacturers, other customers, and independent providers along their purchasing process (Lemon and Verhoef, 2016). In this vein, consumer behavior along the purchasing process has been changing from a linear, single-channel shopping behavior to a complex, network-structured omnichannel behavior that spans over a multitude of different online and offline channels (Srinivasan et al., 2016). In today’s omnichannel retail environment, customers often search in one channel but end up purchasing at another and continuously and often unconsciously switch between the online and the offline world and between different providers (Grewal et al., 2016). This complex and network-structured purchasing process is commonly referred to as the customer journey that encompasses the “customer’s search and purchase usage of all online and offline touchpoints from various sources, including retailers-owned, competitor-owned and additional touchpoints” (Herhausen et al., 2019, p. 11). If customers choose different touchpoints in their search and purchase phase, they engage in so-called research shopping behavior.

Verhoef et al. (2007, p. 129) pioneered the concept of research shopping behavior and defined it as “the propensity of consumers to research the product in one channel and then purchase it through another channel.” Already in 2007, they claimed that searching online and purchasing offline is the most commonly pursued form of research shopping behavior. Nowadays this behavior is referred to as webrooming (Verhoef et al., 2015). Next to webrooming, showrooming behavior is also common, in which consumers first search offline and purchase online (Gensler et al., 2017). According to a recent survey of 2000 shoppers by JRNI (2019), 74% of United States and United Kingdom consumers engage in webrooming behavior, predominantly for electronics, clothing, and household goods. On the other hand, only 57% of shoppers in the United States and United Kingdom engage in showrooming behavior (JRNI, 2019). With more than two thirds of customers researching online before purchasing offline, webrooming is also becoming more and more prevalent in Switzerland and is much more dominant among customers than showrooming (Fuhrer and Hotz, 2018). Not only American and European, but also Asian shoppers are increasingly engaging in webrooming behavior (Nielsen, 2016). A consumer study in nine big cities in Asia yields that almost 80% of these customers engage in both showrooming and webrooming behaviors when purchasing (BusinessToday, 2019).

As webrooming has become common practice in omnichannel consumer behavior across the world (Sands et al., 2016; Herhausen et al., 2019; Flavián et al., 2020), research on the webrooming phenomenon is becoming more and more important. In light of the newly gained complexity that has arisen with the multitude of different touchpoints and providers that customer can choose from along their journey, retailers struggle to identify, understand, and serve webroomers (Flavián et al., 2019). The majority of webroomers was found to engage in free-riding behavior, by searching in one retailer’s channel and purchasing in another retailer’s channel (Heitz-Spahn, 2013). When dealing with free-riders, retailers have to offer their services in the search phase free of charge, but end up missing out on valuable sales that the customer generates in the purchasing phase (Chatterjee, 2010). Therefore, identifying, understanding, and better serving webroomers in order to avoid losing them to competitors in the purchase stage is of utmost importance for omnichannel retailers (Neslin and Shankar, 2009). Still, although showrooming has been studied extensively (e.g., Rapp et al., 2015; Daunt and Harris, 2017; Gu and Tayi, 2017; Gensler et al., 2017; Mehra et al., 2017; Kuksov and Liao, 2018; Fassnacht et al., 2019; Schneider and Zielke, 2020), only a few studies have managed to shed light on the webrooming phenomenon (e.g., Flavián et al., 2016; Arora and Sahney, 2017; Viejo-Fernandez et al., 2018). Therefore, the main objectives of this research work include:

1. Characterizing webroomers and assessing their prevalence in different countries and industries.
2. Assessing the most important antecedents of webrooming behavior in order to better understand the psychological mechanism behind it.
3. Providing first answers to the question on whether webroomers are more valuable to retailers than other customers.

We use data from a large cross-national online survey in which 1497 customers reconstruct a purchase journey in order to differentiate webroomers from pure, online shoppers. Building on the main objectives of this research we will compare webroomers to pure online shoppers and thereby contribute to research and practice in omnichannel retailing in three ways. First, we use data from customers who used retailer-owned, competitor-owned, and independent touchpoints along the search and purchase phase of the customer journey in order to characterize webroomers in an omnichannel context and assess their prevalence in different countries and industries. Our insights help retailers to detect, understand, and serve webroomers in their customer base more easily and increase our understanding of the webrooming phenomenon in an omnichannel retailing environment. Second, we contribute to research on the psychological mechanism behind webrooming behavior by identifying and testing several important antecedents. As described by Verhoef et al. (2020), more research is required on understanding omnichannel consumer behavior. Thirdly, building on the rich literature on the relationship between omnichannel purchase behavior and customer spending (e.g., Kumar and Venkatesan, 2005; Kumar et al., 2018), we use exploratory analyses to provide first insights into the economic value of webrooming behavior.

## Literature Review

Consumers’ perceptions of comparative channel advantages at different stages of the purchase process are the driving force behind research shopping behavior (e.g., Burke, 2002; Ratchford et al., 2003; Frambach et al., 2007). Verhoef et al. (2007) classify channel attributes in terms of benefits and costs and compare online-shops with catalogs and physical stores. They argue that customers who engage in webrooming may benefit the most from comparative channel advantages and find that the Internet is the preferred search channel because it provides fast and easy access to a vast amount of information and thereby facilitates product evaluations (Verhoef et al., 2007). The physical store, on the other hand, is preferred for purchasing due to its enhanced service quality and low purchase risks. Their findings have been consistently confirmed in the literature (Noble et al., 2005; Konuş et al., 2008; Chiu et al., 2011; Avery et al., 2012; Gensler et al., 2012) and thus help us to understand better why webrooming has become so prevalent in today’s omnichannel environment.

Despite the growing importance of webrooming in an omnichannel retail environment, only a few studies examine webrooming in detail. Sands et al. (2016) segment customers based on the importance of four distinct touchpoints (physical store, online store, mobile, and social media) in the search, purchase, and post-purchase stage of the customer journey. They identify three research shopper segments who prefer to research online and purchase offline and thus provide further proof for webrooming as the most prevalent form of research shopping. Flavián et al. (2016) focus on the outcomes of webrooming by examining how the previous interaction with a product online influences customers’ purchase behaviors in the physical store. They find that combining online search and offline purchase for a target product, as compared to search and purchase in-store, increases customers’ purchase intention, search process satisfaction, and choice confidence. In another research work, Flavián et al. (2019) compare webroomers to showroomers and find that webrooming induces smart shopper feelings and confidence in having made the right choice among customers. In turn, these feelings have a positive impact on customers’ search process satisfaction. Another study by Flavián et al. (2020) actively manipulates showrooming and webrooming behavior in an online experiment to assess the influence of these two research shopping behaviors on the customer experience. According to their results, customers think that webroomers are saving more time and effort while shopping than showroomers and that webroomers are more likely to make the right purchase decision than showroomers. Furthermore, the authors find that webrooming behavior is perceived to produce higher levels of control and responsibility among customers than showrooming behavior.

Some existing studies on webrooming identified socio-demographic and psychographic characteristics of webroomers (e.g., Viejo-Fernandez et al., 2018; Flavián et al., 2019) and assessed antecedents of webrooming (e.g., Arora and Sahney; 2017). A conceptual study by Arora and Sahney (2017) integrates the theory of planned behavior and the technology acceptance model to derive propositions on potential drivers of webrooming, such as perceived ease of online search and lack of trust in purchasing online. Viejo-Fernandez et al. (2018) examine more than 4000 customer journeys and find that product attributes are more important for the purchase decision for webroomers than they are for showroomers. Furthermore, they find that most webroomers travel to the physical store with an extensive knowledge on the product and its features and with an already quite concise idea of what they want. Studies on the role of price in research shopping behavior offer mixed results which makes it hard to assess the association between price and webrooming. For instance, studies on antecedents of showrooming show that price is an important factor driving customers to engage in this form of research shopping (e.g., Rapp et al., 2015; Gensler et al., 2017). Interestingly, Viejo-Fernandez et al. (2018) find that showroomers often end up paying higher prices than pure online shoppers for the same product, because they are less sensitive to the product’s price range and more heavily influenced by brands and trendy labels. In this vein, Gensler et al. (2017) also argue that customers decision to engage in showrooming depends on so much more than just on price. Research on the role of price in webrooming behavior is scarce. Some studies argue that price does not seem to be such an important driver of webrooming (e.g., Flavián et al., 2019). By relying solely on existing research, we cannot fully assess whether the factor price plays a role in customers’ decision to engage in webrooming and if yes, how important the price of a product or the price perception of a retailer and its channels is in comparison to other factors.

While all of the above-mentioned studies contribute substantially to our understanding of webrooming, we still lack a comprehensive overview of the prevalence of webrooming behavior across different countries and industries, the characteristics of webroomers in an omnichannel environment, the most important antecedents of webrooming behavior and the value of webrooming for omnichannel retailing. Furthermore, many of the abovementioned studies compare the webrooming behavior with showrooming behavior (e.g., Viejo-Fernandez et al., 2018; Flavián et al., 2019) and thus fail to examine the differences between webroomers and pure online shoppers. Given that engaging in webrooming behavior, i.e., purchasing offline after an online search, is most likely to occur among online shoppers, more studies should compare the characteristics of webroomers and pure online shoppers.

## Background and Hypotheses

**FIGURE 1:**
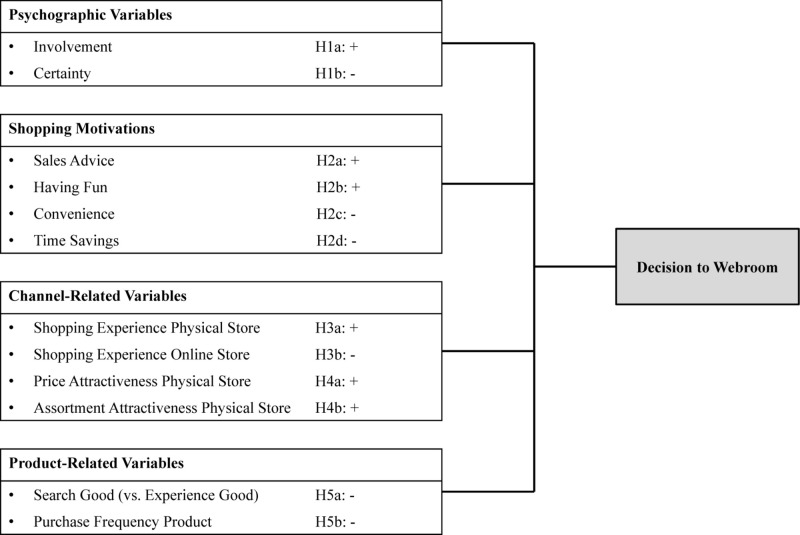
Conceptual model.

Figure 1 shows our conceptual model and the proposed hypotheses. Similar to existing studies regarding research shopping behavior (e.g., Verhoef et al., 2007; Heitz-Spahn, 2013; Gensler et al., 2017), we base our conceptual framework on the antecedents of webrooming on anticipated utility theory (Quiggin, 1982). In line with anticipated utility theory, we consider customers’ decision making along the journey as a forward-looking process in which customers choose to use specific channels depending on their perceived marginal utility. Customers use those channels that best satisfy their individual needs by minimizing costs and maximizing benefits at different stages of the customer journey (Flavián et al., 2016; Gensler et al., 2017). Each channel involves different costs and benefits for the customer that depend on the customer’s psychographic profile, his/her shopping motivations, his/her individual assessment of channel capabilities, and the product that he/she intends to purchase. We expect that customers’ anticipated utility from using the physical store versus the online store for purchase can be predicted by these different variables. We consider four groups of antecedents: psychographic variables, customers’ shopping motivations, channel- and product-related variables (Hypotheses 1–5). Psychographic variables capture all those variables that help to profile customers according to their personality, values, opinions, lifestyles, attitudes, and interests. Customers’ shopping motivations are formed by their individual goals and needs and refer to all the characteristics that are important to customers throughout their entire shopping process and along various channels. Channel-related variables capture customers experience in and assessment of specific channels in the customer journey. Product-related variables refer to different characteristics of products and to customers’ experience in purchasing and/or using these products.

### Psychographic Variables

Product involvement captures the extent to which the product or product category that the customer aims to purchase is personally important to her/him (Petty et al., 1983). High levels of felt product involvement positively affect customers’ attention toward the product and their effort to comprehend it with all its features (Hoffman and Novak, 1996). With the finding that research shoppers place more importance on product-related characteristics (price, features, comparative advantages, etc.) in their journey than single-channel shoppers, Viejo-Fernandez et al. (2018) provide first evidence that research shoppers engage in a more dedicated search than single channel shoppers. Combining online and offline channels along the journey may help customers to benefit from comparative channel advantages and thereby increase the amount of information gathered during search (Verhoef et al., 2007). As a consequence, customers with high levels of involvement are more likely to use different channels in their purchase journey (De Keyser et al., 2015; Herhausen et al., 2019). Given that the customer journey of webroomers allows them to extensively search online and in the physical store before purchase, we expect to find that customers’ level of product involvement is positively associated with their propensity to engage in webrooming.

Customer search behavior also depends on customers’ level of pre-purchase certainty (Urbany et al., 1989). Customers who show high levels of pre-purchase certainty already have a specific idea of what they want to purchase, where they want to purchase, at what price the want to purchase, and which alternatives they can consider (Urbany et al., 1989). The physical and the online store both provide different capabilities that can help customers to decrease uncertainty and make an informed purchase decision, such as touch and feel experiences in the physical store and easy search and comparison opportunities online (Avery et al., 2012). We hypothesize that an extensive search among different channels can help customers to increase their level of certainty with the purchase. Therefore, we expect to find that customers who are more certain about their purchase before starting the customer journey are less in need of the information provided by both online and offline channels and are thus less likely to engage in webrooming.

Based on above-mentioned findings, we hypothesize the following associations between the psychographic variables involvement as well as certainty, and webrooming:

H1: The probability of whether a customer leaves the online shop and purchases in a physical store is (a) positively associated with the customer’s involvement and (b) negatively associated with the customer’s certainty.

### Shopping Motivations

Depending on the different shopping motivations that incite customers to engage in a specific customer journey, some channels may be more attractive to customers than others (e.g., Arnold and Reynolds, 2003; Verhoef et al., 2007; Heitz-Spahn, 2013; Flavián et al., 2020). Research shows that a retailer’s physical store provides customers with much better access to sales advice while shopping than an online store (Alba et al., 1997; Avery et al., 2012). By disseminating product knowledge and creating personalized product bundles, salespeople can increase customers’ confidence and help them to finalize their purchase (Rapp et al., 2015; Gensler et al., 2017). Consequently, customers who place importance on receiving sales advice in their customer journey are more likely to engage in webrooming instead of pure online shopping.

Another shopping motivation that is associated more strongly with the physical store as with the online store is fun. Due to enhanced opportunities to experience products with all five senses and to interact with others while shopping, physical stores are better suited in providing customers with fun shopping experiences (Verhoef et al., 2007; Avery et al., 2012). As multichannel shoppers show relatively high levels of shopping enjoyment (Konuş et al., 2008), one may also assume that the simple act of switching channels and comparing offers across channels may provide customers with fun. Feeling as a smart shopper by combining offline and online channels can induce positive emotions such as pride and excitement among consumers which may also contribute to shopping enjoyment (Schindler, 1989; Flavián et al., 2019). Therefore, we hypothesize that customers who place importance on having fun while shopping are more likely to engage in webrooming than in pure online shopping.

Convenience is one of the most important shopping motivations that is associated with the online store (Avery et al., 2012). Low search costs online provide the customer with vast opportunities to compare products, brands, and prices in a short amount of time (Verhoef et al., 2007). Furthermore, the 24/7 availability of the Internet and the possibility to shop while sitting comfortable at home contribute to the convenience of pure online shopping (Avery et al., 2012). Research on channel switching behavior shows that customers who experienced restrictions on their shopping behavior due to poor traffic connections or unsuitable opening hours in the physical world switch from the physical store to a more convenient channel (Keaveney, 1995). Staying in the online channel for purchase after an online search is considered more convenient for customers as it may help them to reduce physical effort (Verhoef and Langerak, 2001). Thus, we expect to find that high importance of convenience when shopping is negatively associated with webrooming.

In addition to convenience, the goal to get things done quickly in the purchasing process has been found to negatively influence pleasantness and purchase intention in stimulating physical store environments (Holmqvist and Lunardo, 2015). In this sense, Verhoef and Langerak (2001) find that time pressure among consumers positively influences the perceived relative advantage of online shopping. The evidence that the average duration of the customer journey is significantly higher among webroomers than among on pure online shoppers (Herhausen et al., 2019) suggests that webrooming is more time consuming than pure online shopping. Therefore, we assume that customers who place high importance on saving time along their customer journey are less likely to engage in webrooming.

The following hypothesis summarizes the expected associations between the different shopping motivations and customers’ propensity to engage in webrooming:

H2: The probability of whether a customer leaves the online shop and purchases in a physical store is (a) positively associated with the customer’s perceived importance of sales advice, (b) positively associated with the customer’s perceived importance of having fun, (c) negatively associated with the customer’s perceived importance of convenience, and (d) negatively associated with the customer’s perceived importance of time savings.

### Channel-Related Variables

There is a lot of evidence on how customer experience affects retail patronage (e.g., Baker et al., 2002; Verhoef et al., 2009). We transfer the general psychological idea of physical experience enhancing performance in different domains to a retail setting arguing that experience in a physical store enhances consumers’ positive feelings with product purchases in the physical store. Furthermore, psychological research on physical contact shows that even minimal physical contact can increase people’s sense of security and consequently lead them to increased risk-taking behavior (Levav and Argo, 2010). Transferred to a retail context, we propose that physical store experiences lead consumers to a *status quo* buying bias, in which they profit from the benefits of online search, but do not dare to buy online. They feel more secure with the online-to-offline switch due to their prior experiences with the offline store (Eidelman and Crandall, 2014). These prior experiences in the physical store represent the internal standard to buy. Conversely, perceived positive online experiences might produce adaptations in consumers that lead to other internal standards, resulting in a negative influence of the online shopping experience on the channel switch. Therefore, we hypothesize:

H3: The probability of whether a customer leaves the online shop and purchases in a physical store is a) positively associated with the customer’s experience in in-store shopping and b) negatively associated with the customer’s experience in online shopping.

In the context of research shopping, studies found that achieving a low price is especially important for showroomers (e.g., Rapp et al., 2015) whereas the impact of a price advantage in one channel on customers’ propensity to engage in webrooming remains unclear. However, past research on general consumer psychology has shown that the perceived price of a product is unquestionable one of the most important cues utilized during a customer’s purchase decision (Chiang and Dholakia, 2003). In accordance with the positive effect of price attractiveness, perceptions of assortment attractiveness also influence consumer actions (Kahn and Wansink, 2004). Research found that consumers are more likely to purchase from a retail site, if they perceive the variety of the assortment to be greater (Broniarczyk et al., 1998; Townsend and Kahn, 2014; Hunneman et al., 2017). Transferring these insights to the context of webrooming, we hypothesize:

H4: The probability of whether a customer leaves the online shop and purchases in a physical store is positively associated with the perception of (a) higher price attractiveness and (b) higher assortment attractiveness in the physical store compared to the online shop.

### Product-Related Variables

Some studies show that customers’ channel choice and their expectations of the overall customer experience vary depending on the product category that the customer intends to purchase (e.g., Burke, 2002; Van Baal and Dach, 2005; Heitz-Spahn, 2013). Depending on how easy customers can determine product qualities in the search phase of the customer journey, products can be categorized as either search or experience goods (Nelson, 1974). While the qualities of search goods can be evaluated quite well before purchase, experience goods need to be used/consumed first in order to be able to judge their quality and suitability to the customer’s individual needs (Voorfeld et al., 2016). Webroomers typically engage in a relatively extensive search before their purchase by using both online channels and the physical store to carefully research and examine the products (Viejo-Fernandez et al., 2018). For search goods, consumers’ evaluation of products can be very useful to reduce, for example, purchase risk and increase consumers’ purchase confidence (Flavián et al., 2019, 2020). Risk reduction is an important motivation to switch between channels and devices (e.g., De Haan et al., 2018). For experience goods, an extensive search process to learn about attributes is less useful as consumers experience the product after the purchase. Therefore, one would expect to find that webrooming behavior is especially prevalent for search goods and less prevalent for experience goods. Interestingly, in their study on how showrooming and webrooming affect satisfaction and smart shopper feelings Flavián et al. (2019) show that search process satisfaction and smart shopper feelings are higher for experience goods than for search goods among webroomers and showroomers. This might suggest that, in order to optimize these purchase outcomes, consumers should be more likely to engage in webrooming if they purchase experience goods than if they purchase search goods. On the basis of these recent insights, we initially expect that webrooming is less likely to occur for search products than for experience products. However, we acknowledge that there are ample reasons to assume that the relationship might be different.

Building on the above-mentioned discussion, we also expect that customers who engage in habitual purchases where an extensive search across different channels is not necessary any more are less likely to engage in webrooming behavior. In sum, we hypothesize:

H5: The probability of a customer leaving the online shop and purchasing in a physical store is (a) lower if the customer purchases a search good as compared to an experience good and (b) lower if the customer purchases the product frequently.

## Methodology

### Data Collection

Following Lemon and Verhoef’s (2016) recommendation to examine omnichannel behavior along the journey from the customer’s perspective, we collected data via an online customer survey. With the help of an online panel provider, we collected survey data from a stratified sample in three countries: Austria, Germany and Switzerland in autumn 2016. In exchange for a monetary compensation, we asked customers to fill out an online questionnaire and thereby reconstruct their last customer journey that ended with a purchase at a multichannel retailer. We use the same dataset as in Kleinlercher et al. (2018) and Herhausen et al. (2019), but focus on a different set of variables for this research work^1^. In the course of the questionnaire, participants indicated what they had bought, how much they spent, how much time had passed since their purchase, and which touchpoints they used from search to purchase along the journey. To minimize recall bias about the customers’ usage of different touchpoints in the journey, we presented customers with a predefined list of retailer-owned touchpoints (e.g., retailer online shop) and other touchpoints (e.g., competitor online shop). Furthermore, we provided respondents with clear definitions for each touchpoint and conducted several pretests of our list of touchpoints to avoid misperception bias.

From this sample, we excluded customer journeys from the grocery category as grocery journeys differ significantly from other journeys in their relevance of online touchpoints (Nielsen, 2015). Furthermore, we excluded purchases in categories with less than 10 cases (Hair et al., 2014). All the remaining customer journeys in our sample took place in one of nine different categories: apparel, electronics, entertainment, cosmetics, furniture, housewares, sporting goods, craft goods, and toys. Together with groceries, these nine categories represent the most popular multichannel retailers of which customers in Austria, Germany, and Switzerland purchased from in 2016. According to a recent study that evaluates 100 different criteria (such as the amount of omnichannel services offered or the possibilities to interact with the retailer across channels, etc.), the omnichannel maturity of retailers in Austria and Switzerland ranges from 70 to 78% across our nine categories (Handelsverband, 2020; Vesicular Stomatitis Virus [VSV], 2020). To avoid inaccurate statements about the customer’s usage of touchpoints due to recall bias, we excluded all participants where the time interval between the purchase and the survey participation exceeded 3 months. In a next step, we excluded customers who did not search in a retailer’s or a competitor’s online-shop as those are not relevant for our analysis of webroomers and online shoppers (Dahana et al., 2018). Following this logic, we also excluded customers who finalized their purchase at the retailer’s catalog or call center instead of its online-shop or physical store (Dahana et al., 2018). The final sample consists of 1497 (51.5% female, mean age = 41.5 years) customer journeys.

### Measures

With the help of our data on customers’ search and purchase touchpoints along the journey, we divided the sample in two groups of customers: (1) webroomers and (2) online shoppers. We characterized and coded webroomers as those customers who searched in the retailer’s and or a competitor’s online shop but ended up purchasing in the retailer’s physical store. We refer to webroomers who did not search in any competitor’s online shop as loyal webroomers and to webroomers who visited a competitor’s online shop for search as competitive webroomers. We coded those customers as online shoppers who searched in the retailer’s or a competitor’s online shop and purchased at the retailer’s online shop, but never visited the retailer’s or a competitor’s physical store in the course of their journey. Given that all online shoppers search in an online-shop and could potentially be steered to the retailer’s physical store from there, we refer to online shoppers as potential webroomers and compare their attitudes and behavior with those of actual webroomers.

To examine the differences between webroomers and potential webroomers and to identify the antecedents of webrooming, we used a variety of measures which are listed in Table 1. Our measures are mainly single items, as this research is part of a large bi-yearly survey on omnichannel behavior. This survey measures multiple facets of omnichannel behavior and thus the ability to use multiple items per construct is very limited. We measured customers’ shopping motivations with one-item scales based on item batteries from Avery et al. (2012) and Holmqvist and Lunardo (2015) ranging from 1 = totally disagree to 7 = totally agree. To measure shopping certainty, we used a 6-item scale from Urbany et al. (1989) ranging from 1 = very unsure to 7 = very sure. Customer’s involvement was measured on a one-item scale ranging from 1 = not at all important to 7 = very important (Slama and Tashchian, 1985). In order to assess the association between purchasing search versus experience goods with the probability to engage in webrooming, we coded all the categories as containing either search or experience goods according to Nelson (1970; 1974; see Table 1). Finally, we measured customer spending with the amount of money the customers’ indicated as having spent for their purchased product in Euro in order to provide first insights into the monetary value of webrooming.

**TABLE 1:** Operationalization of variables.

**Shopping Experience Online Store** (Novak et al., 2000) How experienced are you in buying [Product X] in online stores? (1 = not experienced at all to 7 = very experienced)
**Shopping Experience Physical Store** (Novak et al., 2000) How experienced are you in buying [Product X] in physical stores? (1 = not experienced at all to 7 = very experienced)
**Purchase Frequency Product** (Hess et al., 2003) How frequently do you buy [Product X]? (1 = not frequently at all to 7 = very frequently)
**Price Attractiveness Physical Store** The prices at the retailer are more attractive… in the retailer’s online shop (1) or in the retailer’s physical store (7)
**Assortment Attractiveness Physical Store** The assortment at the retailer are more attractive… in the retailer’s online shop (1) or in the retailer’s physical store (7)
**Sales Advice** (Avery et al., 2012) How important was it for you to get sales advice at this shopping occasion (1 = not at all important to 7 = very important)
**Having Fun** (Holmqvist and Lunardo, 2015) On this shopping occasion, my primary goal was to have fun (1 = totally disagree to 7 = totally agree)
**Involvement** (Slama and Tashchian, 1985) How important is [Product X] for you? (1 = not important at all to 7 = very important)
**Convenience** (Avery et al., 2012) How important was it for you to shop whenever and wherever you want at this shopping occasion (1 = not at all important to 7 = very important)
**Time Savings** (Holmqvist and Lunardo, 2015) On this shopping occasion, my primary goal was to get things done quickly (1 = totally disagree to 7 = totally agree)
**Certainty** (Urbany et al., 1989) Thinking back to the time when you started seeking information and shopping for [Product X], how sure were you about…? (1 = very unsure to 7 = very sure) … the retailer to shop from … the model to choose … the brand to choose … the features that were available … the performance of the different brands and models … the most important considerations to be used in making the purchase choice
**Search Good (vs. Experience Good)** (Nelson, 1970, 1974) Search goods = 1 (Electronics, entertainment, games, furniture) versus experience goods = 2 (Apparel, sporting goods, cosmetics, houseware, craft goods)
**Customer Spending** Spending for [Product X] measured in Euro

## Results

### Descriptives

58.2% of the examined journeys involved research shopping behavior. 23.0% of customers in the sample engaged in showrooming behavior (*N* = 344), 35.2% were webroomers (*N* = 528), and 41.8% were pure online shoppers or so-called potential webroomers (*N* = 625). Table 2 shows the demographics of our sample and summarizes the most important characteristics of webroomers and online shoppers. We used independent sample *t*-tests to examine whether the mean differences for continuous descriptive variables (e.g., age, household size, etc.) differ significantly between webroomers and online shoppers. We used Pearsons’s chi-squared test to assess the difference between the percentage shares of different values per variable (e.g., Austria, Germany, and Switzerland) across webroomers and online shoppers. With 56.3% (*N* = 297) of webroomers being male, male customers who search online are more likely to purchase in the physical store than female customers (*p* < 0.01). Younger customers who search online are more likely to engage in webrooming than older ones (*M*_Webroomer_ = 41.1 vs. *M*_OnlineShopper_ = 43.1; *p* < 0.05). The duration of the customer journey differs significantly between webroomers and online shoppers (*p* < 0.01). The share of webroomers in purchase journeys that take less than 1 hour is relatively small (22.8%), whereas those journeys that took more than 1 week were predominantly finalized by webroomers (61.2%). The amount of webroomers also differs across countries in the DACH region. The most webroomers among customers who research online can be found in Austria (55%); 44.9% of online researchers purchase in-store in Germany and only 37.3% in Switzerland. The share of webroomers differs also across industries. When purchasing electronics, customers who search online are more likely to finalize their purchase in-store. One reason could be that electronics often involve high investments for customers (e.g., a smartphone or TV) and, thus, customers prefer to try out the products or get personal sales service before purchasing. Similarly, the category furniture is also dominated by a relatively large amount of webroomers (52.9%).

**TABLE 2:** Description of the sample.

	**Webroomers** **Frequency (in%)**	**Pure-Online Shoppers** **Frequency (in%)**	***p*-value** **(χ^2^-test)**
**Gender**			
Male (*N* = 580)	51.2	48.8	0.561
Female (*N* = 573)	40.3	59.7	0.000
**Age groups**			
Millenials (16-34 years; *N* = 411)	51.1	48.9	0.657
Gen X (35-54 years; *N* = 438)	42.5	57.5	0.002
Boomers or older (55+ years; *N* = 304)	43.4	56.6	0.022
**Education**			
Primary School (*N* = 91)	40.7	59.3	0.075
Middle School (*N* = 407)	44.2	55.8	0.020
High School Degree (*N* = 311)	51.8	48.2	0.533
University Degree (*N* = 319)	42.3	57.7	0.006
Other (*N* = 25)	60.0	40.0	0.317
**Duration of the Journey**			
Less than 1 hour (*N* = 320)	22.8	77.2	0.000
Up to 1 day (*N* = 326)	45.7	54.3	0.121
Up to 1 week (*N* = 324)	59.9	40.1	0.000
More than 1 week (*N* = 183)	61.2	38.8	0.002
**Household Size**			
1 person (*N* = 272)	46.0	54.0	0.182
2 persons (*N* = 460)	46.5	53.5	0.136
3 persons (*N* = 188)	47.9	52.1	0.560
4 persons (*N* = 164)	42.1	57.9	0.042
More than 4 persons (*N* = 69)	43.5	56.5	0.297
**Residence**			
Urban (*N* = 657)	50.8	49.2	0.668
Rural (*N* = 496)	39.1	60.9	0.000
**Countries**			
Austria (*N* = 331)	55.0	45.0	0.070
Germany (*N* = 519)	44.9	55.1	0.020
Switzerland (*N* = 303)	37.3	62.7	0.000
**Categories**			
Apparel (*N* = 393)	35.4	64.4	0.000
Electronics (*N* = 339)	59.3	40.7	0.001
Entertainment (*N* = 149)	40.9	59.1	0.027
Cosmetics (*N* = 85)	38.8	61.2	0.039
Furniture (*N* = 51)	52.9	47.1	0.674
Housewares (*N* = 44)	43.2	56.8	0.366
Sporting Goods (*N* = 37)	43.2	56.8	0.411
Craft goods (*N* = 33)	63.3	36.4	0.117
Toys (*N* = 22)	50.0	50.0	1.00

74.6% (*N* = 394) of webroomers engaged in competitive webrooming and 25.4% (*N* = 134) in loyal webrooming. The tendency to engage in competitive rather than loyal webrooming does not differ between men and women (n.s.) and is not a matter of age (*M*_LoyalWebroomer_ = 40.7 vs. *M*_CompWebroomer_ = 41.3; n.s.). The amount of competitive and loyal webroomers differs across the three countries (*p* < 0.05). With almost 38.1% of webroomers who search and purchase at the same retailer, Switzerland has a significantly larger share of loyal webroomers than Germany and Austria where only about one fifth of all webroomers are loyal to a retailer. A comparison of the share of loyal and competitive webroomers across different categories yields a relatively large share of loyal webroomers in apparel (30.9%) as opposed to a relatively small share of loyal webroomers in electronics (18.9%) and entertainment (18.0%).

### Antecedents of Webrooming

In order to identify the antecedents of webrooming behavior, we conducted a binary logistic regression analysis using SPSS. The dependent variable for the logistic regression is a 0/1 indicator of whether the customer engaged in webrooming on her/his focal purchase or not. We entered 13 continuous and one binary predictor (Search Good) into our model. Nagelkerke’s *R*^2^ equals 0.516. The correlations between the independent variables depicted in Table 3 show mostly insignificant or low correlations. Only Shopping Experience Online Store and Shopping Experience Physical Store (0.448) as well as in-store Price and Assortment Attractiveness show significant, moderate correlations (0.309).

**TABLE 3:** Correlation between independent variables.

	**1**	**2**	**3**	**4**	**5**	**6**	**7**	**8**	**9**	**10**	**11**	**12**
1	1											
2	0.244**	1										
3	0.149**	0.019	1									
4	0.068**	0.110**	0.178**	1								
5	0.177**	0.207**	−0.008	0.179**	1							
6	0.097**	0.101**	−0.107**	−0.078**	0.256**	1						
7	0.098**	0.212**	−0.126**	0.118**	0.227**	0.125**	1					
8	0.122**	0.153**	0.080**	0.117**	0.046	0.011	0.448**	1				
9	−0.061^∗^	−0.080**	0.073**	−0.001	−0.101**	−0.060^∗^	−0.135**	0.099**	1			
10	−0.006	−0.072**	0.179**	−0.024	−0.118**	−0.090**	−0.126**	0.072**	0.309**	1		
11	0.093**	−0.008	0.102**	−0.113**	−0.056^∗^	0.096**	0.017	−0.053^∗^	0.003	0.044	1	
12	0.071**	0.281**	−0.093**	0.214**	0.152**	−0.018	0.238**	0.195**	−0.056^∗^	−0.090**	−0.293**	1

(1) Involvement, (2) Certainty, (3) Sales Advice, (4) Having Fun, (5) Convenience, (6) Time Savings, (7) Shopping Experience Online Store, (8) Shopping Experience Physical Store, (9) Price Attractiveness Physical Store, (10) Assortment Attractiveness Physical Store, (11) Search Good (vs. Experience Good), (12) Purchase Frequency Product.** Significant at p < 0.01 (two-tailed).* Significant at p < 0.05 (two-tailed).

Hypothesis 1 stipulated that customers who search online are a) more likely to switch to the physical store for purchase if they show high levels of involvement and b) less likely to switch to the physical store for purchase if they are sure about which product, brand, and retailer to choose. Table 4 shows that the level of involvement is not significantly associated to webrooming. Thus, H1a is not supported. One reason for this finding could be that the multitude of different online channels available today, provides customers with superior opportunities to closely examine products in a pure online environment (Grewal et al., 2016). Blogs and videos of other customers using the product, price comparison portals, and social media posts from friends and family may help involved customers to make an informed purchase decision without necessarily visiting the physical store (Herhausen et al., 2019). In support of H1b, we find that the level of certainty is negatively associated with the propensity to engage in webrooming. This indicates that customers who are certain about the product features, product price, and product brand they want to purchase as well as the retailer they want to purchase it from are less likely to switch from the online shop to the physical store for purchase.

**TABLE 4:** Logistic regression of whether customer engaged in webrooming (1) or pure online shopping (0).

	**Parameter**	**Standard error**	***p*-value**	**Odds ratio**
*Psychographic Variables*				
Involvement	0.038	0.050	0.448	1.039
Certainty	−0.240	0.073	0.001	0.786
*Shopping Motivations*				
Sales Advice	0.569	0.047	0.000	1.767
Having Fun	0.141	0.045	0.002	1.152
Convenience	−0.441	0.053	0.000	0.644
Time Savings	0.044	0.047	0.348	1.045
*Channel-Related Variables*				
Shopping Experience Online Store	−0.321	0.065	0.000	0.726
Shopping Experience Physical Store	0.446	0.071	0.000	1.562
Price Attractiveness Physical Store	0.453	0.080	0.000	1.573
Assortment Attractiveness Physical Store	0.230	0.052	0.000	1.259
*Product-Related Variables*				
Search Good (vs. Experience Good)	0.748	0.168	0.000	2.113
Purchase Frequency Product	0.015	0.039	0.705	1.015
Constant	−2.918	0.619	0.000	0.054

*N = 1151.*

Hypothesis 2 focuses on the antecedents of webrooming that are related to shopping motivations. H2 states that customers who consider (a) sales advice and (b) having fun as important for their shopping occasion are more likely to engage in webrooming whereas customer who consider (c) convenience and (d) time savings as important for their shopping occasion are less likely to engage in webrooming. In support of H2a and H2b, results show that the importance of sales advice and the importance of having fun while shopping are positively associated with customers’ webrooming propensity. According to the odds ratios, getting sales advice is a more important driver of webrooming than having fun (1.767 vs. 1.152). In support of H2c, the importance of convenience when shopping is negatively associated to customers’ propensity to engage in webrooming. Judging from the odds ratio, the convenience benefit of the online store is one of the most important inhibitors of webrooming behavior (0.644). We did not find support on the hypothesis that webrooming behavior is negatively associated to the importance of time savings (H2d). Even though the customer journeys of pure online shoppers have a significantly shorter duration than those of webroomers (see Table 2), the importance of time savings does not seem to be significantly associated to customers’ decision to webroom. One reason for this result could be that a purchase in the physical store may also be associated with time savings. Whereas pure online shopping can save time for customers in the purchasing process (e.g., by saving travel time to the physical store), online shoppers might end up waiting a few days until their purchased product is shipped to their home address (Alba et al., 1997; Avery et al., 2012). The instant gratification that customers receive when shopping in-store may have counteracted the hypothesized negative association between time savings and webrooming.

H3 proposes that customers’ propensity to engage in webrooming is associated with their experience in purchasing online versus offline. In support of H3a and H3b, Table 4 shows that increased shopping experiences in the online store are negatively associated with the decision to webroom, whereas increased shopping experiences in the physical store are positively associated with webrooming. H4 states that customers’ decision to switch from online search to a purchase offline is more likely to occur if customers perceive that the price and the assortment in retailers’ physical stores are more attractive than in their online stores. In support of H4a und H4b, both price and assortment perceptions are associated to webrooming. A comparison of the odds ratios for price attractiveness and assortment attractiveness yields that price advantages constitute a more important lever in fostering webrooming than assortment advantages do (1.573 vs. 1.259). H5 focuses on the product-related antecedents of webrooming behavior. H5a states that customers who purchase search goods are less likely to engage in webrooming than customers who purchase experience goods. In contrast to our hypothesized negative association, we find a positive association between the binary variable Search Goods (vs. Experience Goods) and customers’ propensity to engage in webrooming. Thus, H5a is not supported. If a customer is looking to purchase a search good instead of an experience good, the likelihood that she/he engages in webrooming is more than two times higher. We also did not find support for H5b which hypothesizes a negative association between purchase frequency of the product and the propensity to webroom. This implies that the decision to engage in webrooming versus pure online shopping does not differ significantly between habitual and unique purchases.

### Exploratory Analysis on Webrooming and Customer Spending

There is extensive evidence that purchasing from multiple channels is positively related to customer value (see Kumar et al., 2018 for an overview). Unfortunately, there are no studies on how the monetary value of webroomers would differ from online shoppers. We initially expect that customers who engage in webrooming spend more on their purchases and are thus more valuable than pure online shoppers. Therefore, we conducted and independent samples *t*-test and a hierarchical linear regression analysis to provide first insights into the monetary value of webrooming. The descriptive analysis on spending differences between webroomers and online shoppers yields that webroomers spend an average 27 percent more money on their purchase than online shoppers (*M*_Webroomer_ = 154 vs. *M*_OnlineShopper_ = 121; *p* = 0.024). For our regression analysis, we used customer spending as dependent variable, webrooming (1/0) as the independent variable, and age, gender, education, income, household size, and country as control variables^2^. Table 5 summarizes the results of our regression analysis. Some control variables are significantly associated to customer spending (Model 1). The positive association between gender and customer spending (β = 60.314, *p* = 0.000) indicates that men spend on average more money on their purchases of durable goods than women. One reason for this result could be that men earn about 10–20% more than woman in Germany, Austria, and Switzerland (Bundesamt für Statistik, 2018; Eurostat, 2020). Furthermore, we find that customer spending increases with age (β = 1.035, *p* = 0.026), which may be explained by a higher disposable income among older consumers. The significant positive relationship between webrooming and customer spending (β = 31.90, *p* = 0.024) provides first evidence that webrooming drives customer spending (Model 2). Interestingly, our results show that those purchases where a high amount of money was spent are predominantly finalized by research shoppers. For instance, among the 87 people that spent between EUR 500–2500 for their purchase, 40% engaged in webrooming, 32% in showrooming, and only 28% followed a pure online journey.

**TABLE 5:** Linear regression predicting customer spending.

	**Model 1: Controls**			**Model 2: Main Effect**		
	
	**Parameter**	***SE***	***p*-value**	**Parameter**	***SE***	***p*-value**
Constant	−2.970	44.416	0.947	−22.518	45.169	0.618
**Level 1 Controls**						
Gender	60.314	13.861	0.000	56.706	13.928	0.000
Age	1.035	0.465	0.026	1.102	0.465	0.018
Education	7.309	7.071	0.301	−7.547	7.059	0.285
Household Size	−0.038-	6.706	0.995	0.107	6.694	0.987
Income	1.624	5.107	0.751	2.365	5.108	0.643
Country	9.734	9.487	0.305	12.298	9.538	0.198
**Level 2 Main Effect**						
Webrooming				31.899	14.087	0.024
*R*	0.157			0.170		
*R*^2^	0.024			0.029		
*R*^2^ Change	0.024			0.004		

### Summary

Table 6 provides an overview of the hypotheses tested in our analyses. Eight out of our 12 hypotheses are supported. In order to drive or inhibit webrooming behavior among their customers, retailers may use psychographic variables, shopping motivations, channel-related variables, and product-related variables that are significantly associated with customers’ propensity to engage in webrooming. The importance of receiving sales advice is one of the most important drivers of webrooming whereas the importance of convenience when shopping is one of the most important inhibitors. Furthermore, we find that price advantages are more fruitful than assortment advantages in steering customers from the website to the physical store. Interestingly, we also find that the propensity to engage in webrooming varies across different types of products that customers purchase. When purchasing search goods, such as electronics or entertainment media, customers are more likely to engage in webrooming behavior as opposed to the purchase of experience goods, such as apparel or cosmetics. Concerning the monetary consequences of webrooming behavior, we find that webroomers are more valuable to retailers than pure online shoppers.

**TABLE 6:** Summary of supported and rejected hypotheses.

**Hypotheses**		**Results**	
H1	The probability of whether a customer leaves the online shop and purchases in a physical store is (a) positively associated with the customer’s involvement and (b) negatively associated with the customer’s certainty.	H1a H1b	Rejected Supported
H2	The probability of whether a customer leaves the online shop and purchases in a physical store is (a) positively associated with the customer’s perceived importance of sales advice, (b) positively associated with the customer’s perceived importance of having fun, (c) negatively associated with the customer’s perceived importance of convenience, and (d) negatively associated with the customer’s perceived importance of time savings.	H2a H2b H2c H2d	Supported Supported Supported Rejected
H3	The probability of whether a customer leaves the online shop and purchases in a physical store is (a) positively associated with the customer’s experience in in-store shopping and (b) negatively associated with the customer’s experience in online shopping.	H3a H3b	Supported Supported
H4	The probability of whether a customer leaves the online shop and purchases in a physical store is positively associated with the perception of (a) higher price attractiveness and (b) higher assortment attractiveness in the physical store compared to the online shop.	H4a H4b	Supported Supported
H5	The probability of a customer leaving the online shop and purchasing in a physical store is (a) lower if the customer purchases a search good as compared to an experience good and (b) lower if the customer purchases the product frequently.	H5a H5b	Rejected Rejected

### Robustness Checks

Following a procedure applied by Gensler et al. (2017) to assess the robustness of their results yielded by a logistic regression analysis, we conducted our logistic regression again after we excluded all variables that showed insignificant results in Table 4. With this step we aimed to check whether it is possible that the insignificant predictors may have created significant results for the other predictors in our model (Hair et al., 2014). Excluding the three variables Involvement, Time Savings, and Purchase Frequency Product did not change the significance levels or directions of any of the nine predictors left, which contributes to the robustness our significant associations.

Other studies discuss potential associations between cross-channel consumer behavior and sociodemographic variables such as age, gender, place of residence, employment status, income, and education (e.g., Konuş et al., 2008; Heitz-Spahn, 2013; Herhausen et al., 2019). Based on these studies, one might argue that some sociodemographic variables may be significantly associated to customers’ propensity to engage in webrooming and that the directions and significance levels of our predictors might change if we included sociodemographic variables into our logistic regression model. Therefore, we assessed whether the inclusion of sociodemographic covariates affects the relationships between the different predictors and customers’ propensity to engage in webrooming. We included age, gender, education, residence, household size, and country into our model and re-estimated the logistic regression. We found significant negative associations between webrooming and age, residence, and household size (please see Table 2 for a detailed description on the sociodemographic variables of webroomers and online shoppers). Importantly, adding these six sociodemographic covariates into our model did not change the direction or significance level of our significant variables and only affected their odds ratios marginally. Further information on this analysis can be requested from the authors.

## Discussion

As a result of our limited knowledge on the characteristics of webroomers in an omnichannel environment, as well as the antecedents and monetary outcomes of webrooming, retailers have not yet managed to harness the full potential of this increasingly prevalent phenomenon (Flavián et al., 2019). Given that most webroomers engage in free-riding, retailers often invest in customers in the search phase but miss out on valuable sales that are generated in the purchasing phase (Chatterjee, 2010). In order to increase the likelihood that the customer purchases from their own channels and not from competitor channels, retailers should make it as easy as possible for their customers to switch seamlessly between their online shop and their physical store (Verhoef et al., 2020). Findings from our study shall help research and practice to better identify and understand webroomers in an omnichannel environment and successfully steer them from an online shop to their own physical store. A descriptive analysis of the 625 pure online shoppers in our sample revealed that the chances to successfully turn pure online shoppers into webroomers are quite good as only 11.7% of pure online shoppers indicated that they would by no means purchase in a retailer’s physical store.

Our findings reveal that retailers may use psychographic variables, shopping motivations, channel-related variables, and product-related variables to drive webrooming behavior among customers who research online. We find that sales advice is one of the most important antecedents of webrooming behavior. This is in line with existing research arguing that the possibility to receive personal sales advice is one of the most important advantages of physical store retailing (e.g., Avery et al., 2012). Given that salespeople in the physical store are an important asset to help customers finalize the purchase and induce customer loyalty (Rapp et al., 2015; Fassnacht et al., 2019; Linzmajer et al., 2020), retailers could highlight the possibilities of receiving in-store sales advice in their online channels in order to steer customers who search online to the offline channel for purchase. Note that this can also help to reduce showrooming behavior (Gensler et al., 2017). Furthermore, we find that price advantages in the physical store are more fruitful than assortment advantages in inducing webrooming behavior. Consequently, retailers who aim to increase their share of webroomers could highlight in-store price promotions on their website. Additional analyses from our dataset reveal that 77.3% (*N* = 483) of our pure online shoppers (*N* = 625) could imagine to switch to the physical store for purchase, if they received a price reduction in the physical store. The average amount of price reduction necessary for online shoppers to switch to the physical store is 18.9% of the purchase price.

Retailers who aim to steer online researchers to their physical store with the help of assortment advantages should highlight their in-store product availability and extended offline assortments. We find that 63.4% of pure online shoppers could image to switch to the physical store for purchase if it offered products that are not available in the retailer’s online shop. Similarly, we find that three out of four pure online shoppers could image to switch to the physical store for purchase if it they had their preferred product in stock and thus allowed the customer to take it home right away. We also find that customers’ propensity to engage in webrooming varies across different types of products. Interestingly, contrasting our initial hypothesis which we based on recent findings of Flavián et al. (2019) the probability that customers engage in webrooming is positively associated with purchases concerning search goods. It seems as if customers who aim to obtain goods that can be evaluated well before purchase (i.e., search goods) are more likely to put extra effort in the evaluation of the product in both online and offline channels. On the other hand, customers who aim to purchase experience goods, whose quality cannot be evaluated sufficiently before purchase anyway, seem to choose the easier way of searching and purchasing solely in online channels. Another possible explanation for this result could be that customers feel like returning an unsuitable experience good after having it tried out is easier online (e.g., customers who order apparel online, wear it for several hours without removing the price tag, and eventually return it). When offering search products whose attributes can be judged well (i.e., by searching for product characteristics and customer reports in online channels and then touching and feeling the products in the physical store) retailers are more likely to have a higher share of webroomers in their customer base. Clearly, more research is required, which connects our results with the one of Flavián et al. (2019).

Research argues that steering customers from an online shop to a purchase in the physical store may not only help retailers to prevent free-riding behavior but may also increase cross-selling opportunities and margin advantages in the physical store (Neslin and Shankar, 2009; Rigby, 2014). A recent survey of 2579 customers in Singapore shows that webroomers spend more on their purchase and are more likely to return to department stores as compared to their counterparts without online research activity (Retail Asia, 2020). The economic value of customers who search online but purchase offline is also evident in our finding that webroomers spend more on their purchase than pure online shoppers. Results from our exploratory analysis provide first evidence for a positive association between webrooming and customer spending. This finding is in line with existing research on the superior value of multichannel shoppers (e.g., Kumar and Venkatesan, 2005; Kumar et al., 2018) and points out the potential benefits for retailers who manage to steer customers from search online to purchase in the physical store. Webroomers may also be beneficial to retailers as they can help them to counteract the demise of the physical store. In Europe, the rise of e-commerce forced many physical stores to close down. For instance, since 2005 almost 40’000 retail businesses vanished in Germany and 60–80’000 more are expected to vanish until 2030 (Innovative Fluid Handling [IFH], 2019) whereas 31’000 physical stores have vanished in Switzerland since 2009 (CRIF, 2019). Meanwhile, online retailers are becoming more and more dominant. They are investing heavily in their online shops in order to provide their customers with as many qualities as the physical store provides wherever possible (e.g., chat functions to enable live sales advice or sounds to increase the online shopping experience). Subtly steering online customers to a purchase in the physical store could help multichannel retailers to stand their ground against pure online players and prevent the demise of physical store retailing.

## Limitations

Our research bears some limitations that open up promising avenues for future research. First, our logistic regression analysis relies on survey data that allows us to examine the degree of association between webrooming and several psychographic variables, shopping motivations, channel-related variables, and product-related variables. We hypothesize cause-and-effect relationships between webrooming and the antecedents from these four different categories with the help of existing literature but cannot actually test causality. Future studies should use our correlational results to develop experimental studies which allow to test causality between customers’ propensity to engage in webrooming and its different antecedents. Second, our exploratory research on the monetary value of webrooming provides first evidence for a positive association between webrooming behavior and customer spending. More research is needed to examine whether this association is mainly driven by a relatively small amount of webroomers who engage in very expensive purchases in specific product categories or whether this association holds for all price classes and product categories. Unfortunately, we also could not give any insights into the long-term effects of webrooming on customer satisfaction and loyalty. Even though Flavián et al. (2019) provide some answers to this question by showing that webroomers are more satisfied with their search process than showroomers, many question marks remain. Future studies should examine differences in satisfaction with the purchased product, satisfaction with the entire purchasing process along search, purchase, and post-purchase, and the ultimate effect on customer loyalty between webroomers and pure online shoppers. Third, our study provides a few insights into the differences between loyal and competitive webroomers, but fails to analyze the differences between those two phenomena in-depth. As our study showed that three out of four webroomers engage in competitive behavior and that the share of competitive webroomers varies across countries and industries, more research is needed to better understand the antecedents and consequences of competitive and loyal webrooming. Finally, our results on the positive association between search goods (vs. experience goods) and customers’ webrooming behavior may be considered as counterintuitive if one considers recent findings by Flavián et al. (2019). Future research could investigate customers’ webrooming propensity across different product categories more extensively.

## Data Availability Statement

The datasets presented in this article are not readily available because they cannot be shared without participant’s prior consent. Requests to access the datasets should be directed to KK, kristina.kleinlercher@unisg.ch.

## Ethics Statement

Ethical review and approval was not required for the study on human participants in accordance with the local legislation and institutional requirements. The participants provided their written informed consent to participate in this study.

## Author Contributions

KK, ML, and TR developed the questionnaire and conducted the customer survey. KK analyzed the data and drafted and revised the manuscript. PV and ML revised the manuscript. All the authors contributed to the article and approved the submitted version.

## Conflict of Interest

The authors declare that the research was conducted in the absence of any commercial or financial relationships that could be construed as a potential conflict of interest.

## Footnotes

1 The dataset examined by Kleinlercher et al. (2018) uses customers’ switching behavior from the retailer’s website to its physical store as dependent variable and thus only investigates switching across retailer-owned channels. This research contributes to our understanding of omnichannel consumer behavior as it examines customers’ switching behavior within and across different retailers. As another important difference in the data used, Kleinlercher et al. (2018) use manually coded website characteristics as independent variables whereas this research focuses on customer-reported data from the survey. Herhausen et al. (2019) rely on customers’ touchpoints and several covariates to identify different customer journeys segments with the help of latent class analyses. Even though Herhausen et al. (2019) also identify a webrooming segment, they do not examine variables such as certainty, having fun, sales advice, convenience, price attractiveness, assortment attractiveness or search/experience goods and thus only provide a few insights into the characteristics and antecedents of webrooming.

2 Similar to other studies on shopping expenditures of durable goods (Barigozzi et al., 2012; Schmid et al., 2016), the distributions of spending are right-skewed in our data. Thus, customers spending a disproportionally large amount of money pull the mean spending to a much higher level than the median spending. As a consequence to this distribution in our data, using log(spending) in a second regression analysis did not yield a significant relationship between webrooming and spending.
